# Supplementary material for: Patterns of Intron Gain and Loss in Fungi
Source: PLoS Biol. 2004 Nov 30;2(12):e422. doi: 10.1371/journal.pbio.0020422 (PMC532390; doi:10.1371/journal.pbio.0020422)
Supplement: Table S1 — Also available at http://genes.mit.edu/NielsenEtAl/. (4.3 MB ZIP). [file pbio.0020422.st001.zip › NielsenEtAl/html/1402.html]

AN7673.1.NCU06380.1.MG06060.1.FG05293.1


```
 CLUSTAL W (1.82) Multiple Sequence Alignments - Introns Inserted


Sequence 1: NCU06380.1	531 aa
Sequence 2: FG05293.1	424 aa
Sequence 3: AN7673.1	424 aa
Sequence 4: MG06060.1	413 aa
Alignment Length: 544 aa
Number Identitical Residues: 212 aa
Alignment Score (without introns) 9130


MG06060.1 	-----MPPMRFNHFLALILGVGVAC----TLAGTAPGITVS---------MSLEDLDEKL
NCU06380.1	MAVG----ISRAKCLMMLFALASIAVLQVYARGTHETYGMERKASP--SDMSLEELDGLL
FG05293.1 	MALTRKSVVSTAVCLALLVGLA-------YAGHGHDHHHVETVAGVNIDNMSLEELDTQL
AN7673.1  	-MLFYNTPSARRIGLALIVGIAFFS----ALATAHETFAVG--------ELSVSQIEERL
          	      .       * ::..:.  .              :         .:*:.:::  *

MG06060.1 	Q~QCDVVKEMSAEKAAHFAQTSASSMSALLDMLFPGGPAVNAILATF2YISGPPT~FLLA
NCU06380.1	Q0DCPVVQALSAEKVIAHAHQSSSATERLFAILFPGSPAVNAILATL~YISGPPN~FLLA
FG05293.1 	Q0TCPIVQQLNSVKHAHHAAAPSSLTARAFAVLFPSTPAVNALLATL~YISGPPN~FLLA
AN7673.1  	Q0SCPFVESLNEHKRATSPP-TTSLTSKIFSILFPGSPAVNALLATV~YISGPPN1FLLA
          	*  * .*: :.  *    .  .:*     : :***. *****:***. ******. ****

MG06060.1 	LCPKNIDPSSLSTMVAFAVGGLMGDTLFHLLPEIFLGEDEPDQARFVLVEPNRNLLLGVG
NCU06380.1	LCPTNIDPSSLSVMVAFAVGGLLGDTLFHLLPEIFIGEDSHESAKFVLVEPNRNLLLGLA
FG05293.1 	LCPTNIDPASLSVMVAFAVGGLLGDTLFHLLPEIFVGEDHDAAVKFVLVEPNRNLILGLG
AN7673.1  	LCPPNIDPSSLSVMVAFAVGGLLGDTLFHLLPEIFLGEDSPDHVRFVMVEPNRNLLLGLG
          	*** ****:***.*********:************:***    .:**:*******:**:.

MG06060.1 	ILVGFMTFVAMDKGLRIATGGAGGHDHSHGHSHG-AHAHDATVEVVSATSVDTGKGARSR
NCU06380.1	ILVGFMTFVAMDKGLRIATGGEGGHDHSHGHSHG---GHSHEHGAATGVDVAKKGDAKNR
FG05293.1 	ILVGFMTFVAMDKGLRIATGG-AGHDHSHGHGDAHTHSHSEDKAISSGVDGAD-GAVKSR
AN7673.1  	IMVGFFTFVAMDKALRIATGG-EGHDHSHSHSHS---HSAPETAVATGASTTS-SNTKLK
          	*:***:*******.*******  ******.*...            :...      .: :

MG06060.1 	K-SAGPDKAAANGSKEVATVKEPTQQVNSSGLLNLI2ADFTHNITDGLAMSASFYASPTI
NCU06380.1	KGEKNELKKKDVDAQEEDDKKEVNPSAKLGGYLNLI2ADFTHNITDGLAMSASFYASPTI
FG05293.1 	K-KANEDKGAVVTNAVDKPEKEINPSVKLGGYLNLI2ADFTHNITDGLAMSASFYASPTI
AN7673.1  	Q-RKTAGSHPDIPDT-DSSKNEINPSVKLGGYLNLI~ADFTHNITDGLAMSSSFYASPTI
          	:      .            :* . ..: .* **** **************:********

MG06060.1 	GATTALAV1ALHECPHQVGDFALLIQSGFSKWSAIGLQFVTALGALLGTLIGIAVQEFGG
NCU06380.1	GATTTVAV~FFHEIPHEVGDFALLVQSGFSKRQAMGAQFVTAIGAMLGTLIGIAVQEFGG
FG05293.1 	GATTTVAV~FFHEIPHEVGDFALLIQSGFSKKAAMGSQFITAIGALIGTLIGIAIQEFG-
AN7673.1  	GATTTVAV~FFHEIPHEVGDFALLIQSGFSKRKAMGAQFVTAIGAFLGTLIGIAVQELGG
          	****::**  :** **:*******:******  *:* **:**:**::*******:**:*.

MG06060.1 	SGGEPVLMG------AGLWGTSL~TWGDMLLPFTAGTFLYVGTVAAVPELLETEAGDKVR
NCU06380.1	SSGPSEAA---MGMKEGIWGTSL~TWGDMLLPFTAGTFLYVGTVAVIPELLETGK-DKAK
FG05293.1 	-SPDSDVP---MGRHDGIWGTSL0TWGDMLLPFTAGTFLYVGTVAVIPELLETGP-NKAK
AN7673.1  	SGSPTTADGLASSPVSGLLGTSL~TWGDMLLPFTAGTFLYVGTVSVIPELLETGK-NKSV
          	:.  .   . : .   *: **** ********************:.:******   :*  

MG06060.1 	EVKKTFVQFVAVALGAGIML~YIS-------------WH---------------------
NCU06380.1	ELKKTLVQFAAIAVGAGIML2VKGFVITSGNGNGNRHYHNLNSVLTNLQGHGPSYSGNDH
FG05293.1 	EFKNMIIQFSAVAVGAGIML~YIS-------------WHD--------------------
AN7673.1  	EIKKTIVQFAAVAVGAGIML~ALVP-----------PYKRLVPC----------------
          	*.*: ::** *:*:******                 ::   .                 

MG06060.1 	----------------------------------------------~-------------
NCU06380.1	SHQAHIYAPSQVLLGQAMSSAPKDRHKHREKSVTPRYKQPWCQSIM0GRRCFTVESNWAV
FG05293.1 	----------------------------------------------~-------------
AN7673.1  	----------------------------------------------~-------------
          	                                                            

MG06060.1 	------------
NCU06380.1	FTTEFKTPYRML
FG05293.1 	------------
AN7673.1  	------------
          	
```
